# Application of the Appropriate Use Criteria for Coronary Revascularization in Patients with Acute Coronary Syndrome in the Russian Federation: Data from the Federal Registry

**DOI:** 10.5152/eurasianjmed.2021.20004

**Published:** 2021-06

**Authors:** Yuliya V. Popova, Anton R. Kiselev, Olesya V. Sagaydak, Olga M. Posnenkova, Vladimir I. Gridnev, Elena V. Oshchepkova

**Affiliations:** 1 Saratov State Medical University, Saratov, Russia; 2 National Medical Research Center of Cardiology, Moscow, Russia

**Keywords:** acute coronary syndrome, percutaneous coronary intervention, registry

## Abstract

**Objective:**

The aim of the study was to apply the appropriate use criteria (AUC) for coronary revascularization on Russian Acute Coronary Syndrome Registry (RusACSR) data to analyze validity of the decision to perform percutaneous coronary interventions (PCIs) among patients with acute coronary syndrome (ACS).

**Material and Methods:**

In Russia, the frequency of performing PCI increased almost 7.5 times, and more than half of all interventions were performed in patients with ACS, in the period from 2006 to 2015. AUC 2012 were used to assess PCI appropriateness. Data were exported from RusACSR from a period of January 1, 2016 to December 31, 2016. We analyzed 33 893 cases, but 13 957 patients were excluded owing to absence of data needed. The study group therefore included 19 936 patients with ACS (mean age, 65.3 ± 11.9 years; 40.3% women), and it was divided into 2 subgroups: 13 757 (67.2%) patients who were treated conservatively and 6179 (32.8%) patients who underwent PCI. According to AUC, physicians’ choice of strategy was validated.

**Results:**

Patients treated conservatively differed significantly (*P* < .001) from those who underwent PCI. In this group, non-ST segment elevation ACS was significantly more common than in the group of patients who received PCI (84.4% vs. 43.9%, *P* < .001). They also had more severe clinical status. According to AUC, among patients with ACS treated with PCI, the decision was warranted in 86.3% (valid decision). In 7.6% of cases, there was no need for PCI. Among patients who underwent conservative treatment, 77.7% of patients needed PCI according to AUC. According to our data, only 3.8% of patients who were treated conservatively did not need PCI. Appropriateness of invasive treatment was uncertain in 18.5% and 6.1% in the PCI and non-PCI groups, respectively. All differences were significant (*P* < .001).

**Conclusion:**

AUC implementation showed low availability of PCI for patients with non-ST segment elevation ACS accompanied by complicated clinical status. AUC for coronary revascularization could be applied in Russian clinical practice for unbiased PCI candidate selection and for evaluation of decision validity.

## Introduction

In the Russian Federation, the frequency of performing percutaneous coronary interventions (PCIs) is far from the ideal and is much less than in developed countries. However, the situation is improving; in the period from 2006 to 2015, the frequency of performing PCI increased almost 7.5 times and more than half of all interventions are performed in patients with acute coronary syndrome (ACS).^1^

PCI is a gold standard for treating patients with ACS with ST segment elevation on electrocardiogram (STE-ACS). According to current clinical guidelines, it is necessary to strive to perform this procedure in as many patients as possible.^2^ According to data from the Russian Acute Coronary Syndrome Registry (RusACSR), in 2015 in different regions of Russia, the proportion of patients with STE-ACS who underwent PCI ranged from 4.8% to 88.1%.^3^ However, in relation to primary PCI, Russia lags behind European countries: in 2015, the frequency of primary PCI in Russia was 317 per 1 million population, whereas in Europe in 2010–2011, the frequency of primary PCI ranged from 460 to 804 per 1 million population.^1^

Thus, a significant number of patients with ACS in Russia do not receive proper medical care. This is due to both the lack of well-organized healthcare infrastructure in some regions and problems in making a medical decision and evaluating indications for PCI. Therefore, it seems necessary to create a unified expert assessment system that would allow an objective opinion about the appropriateness of performing interventions on coronary arteries in case of ACS in real clinical practice. This will allow for identifying patients with absolute indications for PCI and patients for whom intervention is not needed according to the evidence-based medicine data, and it will allow for effective distribution of the costs of the healthcare system.

There is a large amount of data collected from all around the world on PCI in patients with various forms of ACS. Based on accumulated evidence, recommendations and guidelines from the leading professional communities were developed, such as guidelines of the European Society of Cardiology for managing patients with elevation^2^ and without elevation of the ST segment on electrocardiogram (ECG),^4^ guidelines of the European Society of Cardiology for myocardial revascularization,^5^ guidelines of the American College of Cardiology for PCIs,^6^ and Russian national guidelines of the Russian Acute Cardiovascular Care Association.^7^ However, the provisions of these guidelines are not always fully implemented in clinical practice in patients with ACS, which lowers the quality of medical care provided. There are a number of tools to assess the quality of medical care and its compliance with the approved clinical guidelines and standards. One of these tools is the Appropriate Use Criteria for Coronary Revascularization (hereinafter referred to as the Criteria) developed by the American College of Cardiology.^8^

The Criteria serve as a supplement to the practical guidelines of the American College of Cardiology to assess the appropriateness of using coronary revascularization procedures in various clinical situations^8^ and are a list of the most typical clinical and anatomical cases in patients undergoing myocardial revascularization, as well as clinical decisions on the need for coronary revascularization in each individual case. The Criteria also make it possible to assess the need for PCI in those patients with ACS in whom PCI was not performed.

To assess the appropriateness of the decisions made about whether there was a need for PCI for patients with ACS in this study, the Criteria were applied to patients from the RusACSR database. The study objective was to apply the Appropriate Use Criteria for Coronary Revascularization to determine the appropriateness of performed PCI and the need for this type of intervention in patients included in RusACSR.

## Materials and Methods

### Data source

Data of patients with ACS were exported from RusACSR.^9^ RusACSR is a multicenter retrospective registry that contains demographic data, clinical characteristics, and information about treatment (including reperfusion therapy) of patients with ACS who were admitted and received treatment in hospitals of the Russian Federation.

RusACSR is filled at the inpatient treatment stage and is a computer system that allows for the accumulation and analysis of data on patients with ACS older than 18 years of age. The data are de-identified, encrypted, and stored only with the patient’s consent (the patient signs an informed consent). In accordance with federal laws, the data are transferred to the server of the National Medical Research Center of Cardiology (Moscow, Russia) in anonymized form via a certified secure channel. Data are exported using remote server access via website interface.^10^

The Ethics Committee of Saratov State Medical University in Saratov, Russia, approved the study (Protocol No. 1, 5 February 2019).

RusACSR has been operating since 2008. Since then, data on more than 390 000 patients with ACS were collected from 62 regions of the Russian Federation (74% of all 85 regions).

### Appropriate use criteria for coronary revascularization

United States Appropriate Use Criteria for Coronary Revascularization, 2012 (the Criteria)^8^ were used to assess PCI appropriateness. The Criteria represent different clinical scenarios. Scenarios are a combination of clinical characteristics of patients with ACS, including the following:

- Diagnosis on admission,
- Clinical status on admission (heart failure, hemodynamic or electrical instability, recurrent ischemia, left ventricular ejection fraction),
- Time from symptom onset to PCI,
- Data on the success of previous thrombolysis,
- Number of affected coronary arteries, and
- Risk of death or nonfatal myocardial infarction in the near future.

For the Criteria, experts have developed opinions on the appropriateness of myocardial revascularization based on the clinical guidelines for each of the clinical scenarios; revascularization is appropriate, inappropriate, or questionable. This study used the Criteria published in 2012, scenarios no. 1–13.^8^

### Patient data

In this study, we analyzed data of 33 893 patients with ACS uploaded into the RusACSR system in the period from January 1, 2016 to December 31, 2016.

In the study, 19 936 (58.8%) patients had all the clinical data necessary for the application of the Criteria (59.7% males; age, 65.3 ± 11.9 years, presented as mean with standard deviation). A total of 13 957 (41.2%) patients had insufficient clinical data to apply the Criteria, and therefore, these patients were excluded from the study. Patients included in the study were divided into 2 groups: patients who underwent PCI (n = 6179, 31% of the total group) and patients who were not treated with PCI (n = 13 757, 69% of the total group).

After applying the Criteria on patients who underwent PCI, it was determined whether PCI was appropriate, not appropriate, or questionable. Appropriateness assessment was carried out only for the first PCI performed during hospitalization.

The need for PCI among patients with ACS who received conservative treatment (meaning no PCI was performed) was also assessed. Groups of patients who had the need for PCI, who had no need for PCI, and for whom the need for PCI was doubtful were identified.

### Statistical analysis

Data processing was performed by using STATISTICA 6.1 software package (StatSoft^®^, USA). Quantitative data are presented as mean and standard deviation and binary (such as yes/no) variables as frequencies in absolute values and in percentage. Frequencies between the groups of patients were compared on the basis of the chi-square test. Reliability of the used statistical evaluations was taken as at least 95%.

## Results

### Clinical patient profile

Table 1 presents comparisons of clinical profiles of patients with ACS who were included versus excluded from the study and patients with ACS who underwent and did not undergo PCI. Included and excluded patients differed significantly in the majority of clinical traits; however, it seemed to be more statistical than clinical differences.

The reasons for exclusion are presented in Table 2. The most common reason was the absence of corresponding appropriate use criteria (44.9% of excluded patients). This reason included patients both with non-significant stenosis (<50% in the left main and <70% in the other coronary arteries) and the presence of other significant coronary artery stenosis except those noted in the clinical scenario. Absence of coronary angiography data was the second most common reason for exclusion (32.1%). The third reason among 12.9% of excluded STE-ACS patients was the absence of date and time of symptom onset.

Patients treated conservatively differed significantly (*P* < .001) from those who underwent PCI. Analysis showed that among patients who were treated conservatively, there were significantly more women (45.9% vs. 27.9%, *P* < .001) and these patients were older (age in the conservative group was 66.9 ± 11.9 years vs. 61.6 ± 11.1 years in the PCI group; *P* < .001). In this group, ACS without ST elevation on ECG (NSTE-ACS) was significantly more common than in the group of patients who received PCI (84.4% vs. 43.9%, respectively; *P* < .001). That correlates with a significantly more severe clinical status of patients with conservative treatment as compared with patients who underwent PCI; they appeared to have a higher rate of chronic heart failure (CHF) (49.8% vs. 32.0%, *P* < .001), reduced left ventricular ejection fraction (20.6% vs. 13.2%, *P* < .001), and history of myocardial infarction (27.5% vs. 18.1%, *P* < .001) (Table 1).

### Evaluation of PCI appropriateness among patients with acs who underwent revascularization

**Figure 1. a, b:**
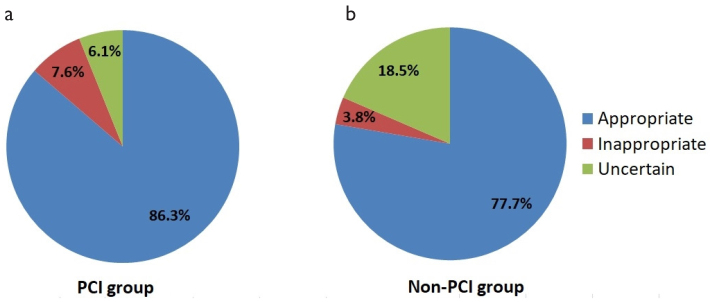
(a) PCI appropriateness in PCI group and (b) non-PCI group. PCI, percutaneous coronary intervention.

Analysis of the group of patients with ACS who underwent PCI showed that according to the Criteria, 5332 interventions out of 6179 (86.3%) were appropriate (Figure 1A). Of these interventions, half (49.7%) were performed in patients with STE-ACS during the first 12 hours from symptom onset.

Only 467 (7.6%) interventions were not appropriate according to the Criteria. These were patients with STE-ACS who underwent PCI after more than 12 hours from symptom onset or with no symptoms on admission and no hemodynamic and electrical instability.

The appropriateness of 380 (6.1%) procedures was determined as doubtful. Most of these patients (87%) were NSTE-ACS with a low risk of death and adverse cardiovascular events.

### Assessment of the need for PCI among patients with ACS who received non-surgical treatment (conservative treatment)

Among 13 757 patients with ACS who were treated conservatively, 10 695 (77.7%) needed PCI according to the Criteria (Figure 1B). Most of these patients (59.7%) were patients with NSTE-ACS and a high risk of death and adverse cardiovascular events.

PCI was not needed only in 516 (3.8%) patients treated conservatively. These were patients with STE-ACS who presented in the hospital after 12 hours from symptom onset, had no symptoms on admission, and did not have hemodynamic and electrical instability.

In 2546 (18.5%) patients, the need for PCI was questionable according to the Criteria. These patients were diagnosed with NSTE-ACS and a low risk of death and adverse cardiovascular events.

In the non-PCI group, patients with ACS that required invasive treatment were significantly rare as compared with the PCI group (77.7% vs. 86.3%, *P* < .001), and patients who did not need PCI were half as common (3.8% vs. 7.6%, *P* < .001). The proportion of patients with ACS in whom PCI appropriateness was uncertain was 3 times higher in the non-PCI group (18.5% vs. 6.1%, *P* < .001). These comparisons are shown in Figure 1.

## Discussion

In daily practice, to estimate the individual need for PCI in patients with ACS, doctors refer to clinical guidelines. In Russia, guidelines of the European Society of Cardiology^5^ are used, as well as national guidelines of the Acute Cardiovascular Care Association.^11^ In other countries, such as the United States (USA) and Japan, physicians use the Criteria.^8^,^12^ The Criteria are based on the current clinical guidelines, but in addition to recommendations, they contain tools for everyday clinical practice and a detailed description of a number of typical clinical situations. For example, the Criteria describe in detail different situations for patients with STE-ACS who underwent thrombolysis; for patients with NSTE-ACS, the Criteria take into consideration the type of revascularization (monovascular or multivascular). However, for the latter category of patients, the Criteria do not indicate the PCI recommended time, whereas the clinical guidelines indicate the time frame for the intervention. Thus, the Criteria and clinical guidelines complement each other and it is advisable to integrate the provisions of both documents into practical medicine. In the Russian Federation, doctors widely use clinical guidelines, whereas the Criteria are not widely applied. This study demonstrates the results and benefits of applying the Criteria in the Russian population of patients with ACS.

It has been shown that according to RusACSR, most PCI in patients with ACS can be considered appropriate, according to the Criteria (86.3%). Only 7.6% of the procedures were recognized as inappropriate. For some patients with ACS from the analyzed group, it was impossible to determine the appropriateness of a medical decision using the Criteria because of the lack of necessary clinical data (time of pain syndrome onset, time when coronary revascularization started, results of coronary angiography, ECG data, information about thrombolysis success). This indicates a lack of diagnostic justification in some decisions made about the necessity of PCI in patients with ACS and also indirectly indicates insufficient control over the quality of medical care for such patients.

First results of the Criteria application were published in 2011 in the USA. Data of the National Cardiovascular Data Registry (USA) showed that among 355 417 patients with ACS, the majority (98.6%) of PCIs were considered reasonable; in 0.3% of patients with ACS, PCI indications were considered doubtful, and as few as 1.1% of cases were assessed as unreasonable PCI. However, the proportion of patients with ACS who have insufficient clinical data was <1%.^13^ Similar data were demonstrated in the study by Bradley,^14^ where the following results were obtained (data from Washington State, USA): among all PCIs performed in 2010 in patients with ACS, 98% of interventions were appropriate, 1% were not appropriate, and <1% were doubtful. In this study, it was not possible to apply the Criteria because of a lack of data in 15% of patients with ACS. According to the Japanese Cardiovascular Database PCI (JCD-PCI) Registry, from 2008 to 2013, in 77.6% of patients with ACS out of 5100, PCI was appropriate; in 19.5% of patients, PCI was doubtful; and in 2.9% of ACS cases, PCI was inappropriate. Among all 11 258 patients included in the JCD-PCI Registry (ACS and stable coronary artery disease), only 182 lacked the clinical data necessary to determine the appropriateness of PCI.^15^

Compared with these results, the proportion of appropriate PCIs in Russia is quite high. The frequency of inappropriate coronary revascularization procedures in patients with ACS in the Russian Federation exceeded that in developed countries. It is also noteworthy that in Russia, the proportion of patients with not enough data to determine the appropriateness of revascularization in case of ACS is significantly higher than in mentioned studies.

One of the most important parts of this study was the assessment of the proportion of patients with ACS who needed PCI among those who were treated conservatively. The results of the study showed that in 2016, among patients whose data were uploaded into RusACSR and who were treated without PCI, 10 695 (77.7%) patients did not receive the care they needed, which corresponds to more than half of all patients with ACS selected for the study (53.6%). There were only a few patients who had no need for PCI (3.8%) among patients with conservative treatment chosen. Among those with ACS who did not receive PCI, there were more females, these patients were older than patients who had PCI, and a history of cardiovascular diseases and diabetes mellitus was more common in these patients. Similar data were obtained by the authors in an earlier study.^16^ Sabouret et al.^17^ presented similar results regarding the age and gender of patients with ACS who had undergone and not undergone PCI. The study by Udell^18^ found that among patients with ACS who did not undergo PCI, there were more women and people suffering from hypertension and CHF. These studies demonstrate the current trend: rarer performance of PCI in women, elderly patients, and those with severe clinical status.

This tendency may lead to an increased mortality of patients with ACS and an increase in the incidence of complications. Therefore, there is obviously a need for an objective estimation tool that helps to make a clinical decision on the necessity of PCI, based on determined indications for this intervention.

This study is very important for low- and middle-income countries with limited PCI availability. Rating of patients’ appropriateness for PCI using universal methodology coupled with information technology may substantially support decisions on treatment strategy and facilitate reimbursement as well as quality assessment. Patients with appropriate PCI will be offered priority in invasive treatment. Monitoring of the population level of PCI appropriateness could drive establishment of PCI hospitals in the regions with the highest number of potentially appropriate patients.

In conclusion, the results of the use of the Criteria demonstrated that this tool can be used in the Russian population of patients with ACS. According to the Criteria applied on the data from RusACSR, in Russia, PCI was appropriate in most patients with ACS. Among patients who received non-surgical treatment, more than 70% had objective indications for PCI, but the intervention was not carried out.

Moreover, implementation of the Criteria showed low availability of PCI for patients with NSTE-ACS accompanied by complicated clinical status.

The Criteria may complement existing clinical guidelines. The use of these two types of guidelines, both at the patient level to determine individual need and appropriateness and at the group level, can increase objectivity in deciding whether to perform PCI. Introduction of the Criteria in the automated module of RusACSR can help in selecting candidates for coronary revascularization and avoid unreasonable procedures, as well as retrospectively help to form an opinion on the appropriateness of the interventions that have already been performed.

**Main Points**

- In Russia percutaneous coronary intervention (PCI) was appropriate in most patients (86.3%) with acute coronary syndrome (ACS).
- 77.7% of ACS patients with conservative treatment needed PCI.
- 3.8% of ACS patients who were treated conservatively actually didn’t need PCI.
- In 18.5% of ACS patients with conservative treatment the need for PCI was questionable.

## Figures and Tables

**Table 1:** Clinical Profile of Patients with ACS

Parameters	Excluded (n = 13 957)	Included, total group (n = 19 936)	*P*-value	PCI group (n = 6179)	NPCI group (n = 13 757)	*P*-value^a^
Male	8871 (63.6%)	11 893 (59.7%)	<.001	4456 (72.1%)	7437 (54.1%)	<0.001
Age, years	65.2 ± 10.3	65.3 ± 11.9	.421	61.6 ± 11.1	66.9 ± 11.9	<0.001
STE-ACS	4937 (35.4%)	5611 (28.1%)	<.001	3464 (56.1%)	2147 (15.6%)	<0.001
NSTE-ACS	9020 (64.6%)	14 325 (71.9%)	<.001	2711 (43.9%)	11 610 (84.4%)	<0.001
History of MI	3723 (26.7%)	4903 (24.6%)	<.001	1120 (18.1%)	3783 (27.5%)	<0.001
History of PCI	1060 (7.6%)	1396 (7.0%)	.036	521 (8.4%)	875 (6.4%)	<0.001
Hypertension	10 472 (75.0%)	16 848 (84.5%)	<.001	5182 (83.9%)	11 666 (84.8%)	<0.001
Type 2 diabetes	2289 (16.4%)	3415 (17.1%)	.089	914 (14.8%)	2501 (18.2%)	<0.001
CHF	5895 (42.2%)	8826 (44.3%)	<.001	1977 (32.0%)	6849 (49.8%)	<0.001
LVEF <40%	3400 (24.4%)	3646 (18.3%)	<.001	816 (13.2%)	2830 (20.6%)	<0.001
Systolic BP	137 ± 24	138 ± 22	<.001	137 ± 23	138 ± 21	0.003
Diastolic BP	83 ± 16	82 ± 15	<.001	82 ± 17	82 ± 19	1.000
Killip class III	328 (2.4%)	473 (2.4%)	1.000	71 (1.1%)	402 (2.9%)	<0.001
Killip class IV	57 (0.4%)	492 (2.5%)	<.001	210 (3.4%)	282 (2.0%)	<0.001
Admission to PCI hospital	6685 (47.9%)	10 486 (52.6%)	<.001	6179 (100%)	4307 (31.3%)	<0.001
In-hospital mortality	516 (3.7%)	518 (2.6%)	<.001	111 (1.8%)	399 (2.9%)	<0.001

Quantitative data are presented as mean ± standard deviation, binary variables as n (%).

ACS, acute coronary syndrome; CHF, chronic heart failure; LVEF, left ventricular ejection fraction; MI, myocardial infarction; NPCI, patients who were not treated with PCI; NSTE-ACS, non-ST segment elevation–acute coronary syndrome; PCI, percutaneous coronary intervention; STE-ACS, ST segment elevation–acute coronary syndrome.

a Comparison by chi-square test of PCI group with NPCI group.

**Table 2:** Reasons for Exclusion (n = 13 957)

Reason for exclusion	n	%
Absence of date and time of symptom onset in STE-ACS patients	1800	12.9
Absence of date and time of PCI	614	4.4
Absence of coronary angiography data	4480	32.1
Absence of data on success of fibrinolysis	530	3.8
Absence of data on LVEF in patients with successful fibrinolysis	265	1.9
Patient angiography data did not correspond with Criteria	6267	44.9

LVEF, left ventricular ejection fraction; PCI, percutaneous coronary intervention; STE-ACS, ST segment elevation–acute coronary syndrome.
